# Patient-elected low-dose intravenous naloxone for rapid buprenorphine induction: a case report

**DOI:** 10.1186/s13722-025-00572-z

**Published:** 2025-05-14

**Authors:** Pouya Azar, Jessica Machado, Jane J. Kim, James S. H. Wong, Mohammadali Nikoo, Victor W. Li

**Affiliations:** 1 Complex Pain and Addiction Service, Vancouver General Hospital, Vancouver, BC Canada; 2 Department of Psychiatry, University of British Columbia, Vancouver, BC Canada

**Keywords:** Buprenorphine, Induction, Naloxone, Opioid use disorder, Opioid agonist therapy

## Abstract

**Background:**

Buprenorphine is a common partial opioid agonist treatment for opioid use disorder (OUD). Despite its efficacy, major challenges to induction include the significant time consumption and the difficult requirement for patients to be in moderate opioid withdrawal.

**Case presentation:**

We present the case of a 31-year-old man with severe OUD and regular fentanyl use who was successfully initiated on buprenorphine-naloxone using low-dose intravenous naloxone in ten minutes and administered 300 mg of extended-release injectable buprenorphine within two hours. This involved the rapid administration of small doses of intravenous naloxone with an assessment of withdrawal symptoms after each dose. Buprenorphine-naloxone is immediately administered once moderate withdrawal is reached.

**Conclusions:**

Low-dose intravenous naloxone provides an alternative method of buprenorphine induction that limits the experience of withdrawal to a shorter time window compared to existing protocols.

## Background

Opioid overdose deaths in North America have reached unprecedented levels over the past two decades. In Canada, a total of 47,162 opioid toxicity deaths were reported between January 2016 and March 2024 [1]. The province of British Columbia is one of the most severely impacted by the crisis, with 41 deaths per 100,000 population in 2024 [2]. Buprenorphine is a partial mu-opioid receptor agonist and a common medication provided as opioid agonist treatment (OAT) for opioid use disorder (OUD). Due to its high binding affinity, it carries the risk of precipitated withdrawal if introduced in the presence of a full-agonist opioid such as fentanyl or heroin. Precipitated withdrawal is characterized as a withdrawal state that comes by the administration of either a partial agonist like buprenorphine, or a full antagonist like naloxone [3]. To avoid precipitated withdrawal, patients must abstain from use and be in at least mild opioid withdrawal (Clinical Opioid Withdrawal Scale [COWS] score of at least 8 to 10) prior to buprenorphine induction [4–6]. Despite these precautions, the rapid proliferation of fentanyl and analogues in the unregulated drug supply commonly complicates the induction process due to precipitated withdrawal increasingly reported in those with recent fentanyl use [7, 8]. This prerequisite of withdrawal can be both time-consuming and challenging for patients, and a common reason that patients do not complete induction [9]. Additionally, waiting for a patient to enter withdrawal can require long periods of monitoring that are often undesirable in the emergency department (ED) and other unobserved settings.

Alternative protocols such as low-dose and high-dose inductions have been employed to address this patient concern. Low-dose (i.e., “microdose”) inductions involve the overlapping induction of buprenorphine in small escalating doses while continuing or tapering full agonist opioids [10]. They are increasingly being used as an induction strategy that reduces the risk of precipitated withdrawal and does not require the patient to abstain from opioids. However, most low-dose approaches can take at least 5 to 7 days to reach a therapeutic dose [11], by which patients are often lost to follow-up. High-dose (i.e., “macrodose”) inductions start with an initial dose of buprenorphine up to 32 mg to achieve therapeutic levels in less than 3 to 4 h [12]. Evidence through a large case series has shown the usefulness of high-dose buprenorphine administered in the ED [13, 14] as well as in pre-hospital settings at the point of overdose [15, 16]. However, not all patients are suited to receive a high-dose induction, such as those are more ill and require hospitalization [12]. Another variation of the high-dose protocol includes a rapid 1-day induction to injectable buprenorphine, which was also found to be feasible and well-tolerated in patients using fentanyl and fentanyl analogs [17].

A few other groups have described using a “rescue” approach of using buprenorphine to initiate treatment after a naloxone-induced opioid withdrawal [18, 19]. In one case report, 0.5 mg of intravenous (IV) naloxone was used to bring about elective withdrawal, which resulted in an increase of COWS score from 0 to 17 in 5 min [19]. This was followed by 4 mg of sublingual buprenorphine with 8 mg of IV ondansetron for symptomatic control. The patient reported feeling well 105 min after naloxone administration and was discharged with 8 mg of sublingual buprenorphine daily. Another case report used 4 mg of intranasal naloxone, self-administered by the patient at home over telemedicine, followed by 24 mg of buprenorphine [18]. Limited evidence suggests that patients can be rapidly inducted onto buprenorphine after a deliberately induced withdrawal with naloxone. Given the impracticality of waiting for spontaneous withdrawal, we propose to incrementally advance COWS scores through IV naloxone administration until a threshold until patients experience moderate withdrawal and a standard dose of buprenorphine can be administered. In particular, our goals were to limit the severity of withdrawal that patients experience when administered full treatment doses of naloxone and further shorten the overall induction time. We present a case of a patient with severe OUD who was successfully inducted onto buprenorphine-naloxone and then extended-release injectable buprenorphine with this novel protocol of using small and incremental doses of naloxone to induce adequate withdrawal. Written consent from the patient was obtained for both participation and publication of this case report.

## Case presentation

The client is a 31-year-old male with severe OUD who was admitted to hospital after being found down and unresponsive to naloxone administration with aspiration pneumonia. Due to the patient’s lack of response to naloxone and a urine drug screen (UDS) that was positive for benzodiazepines, as well as methamphetamines, cannabinoids, cocaine, and fentanyl, it was therefore thought to be a potential benzodiazepine overdose. He remained quite drowsy for a period of three days in hospital. In the interim, our team provided supportive medications including as needed hydromorphone (8–16 mg Q2H) for any emergent symptoms of withdrawal. Clinical Institute Withdrawal Assessment (CIWA) was ordered with as needed diazepam (10–20 mg PO/IV Q1H) given the suspected benzodiazepine use. His aspiration pneumonia was treated with 500 mg of oral azithromycin for three days and 2000 mg of intravenous co-trimoxazole for four days. The patient became more alert by the third day in hospital.

Our team then collected a comprehensive history from the patient. He did not have any diagnosed psychiatric or medical illnesses and was living in a single resident occupancy (SRO) hotel. He reported supporting himself financially through the unregulated drug trade. His substance use history included OUD and stimulant use disorder. He self-reported smoking 1 to 1.5 g of unregulated fentanyl daily and having suffered from two overdoses responsive to naloxone in the past with associated ED visits. He has previously been treated with a 5-day micro-induction of buprenorphine-naloxone as an outpatient but only completed 3 days due to feelings of withdrawal. He also had been treated with sustained oral release morphine (SROM) with a dose of 200 mg daily for a period of a couple of days and 40 mg methadone daily for less than a week, with unknown and undisclosed reasons of stopping these medications. He was not on any OAT at the time of presentation to the hospital. He also reported using 1 to 1.5 g of methamphetamine daily for years. The patient did not want to stay any longer in hospital but was interested in being restarted on OAT. OAT options were provided to the patient, and he ultimately preferred receiving the buprenorphine depot injection. The low-dose intravenous naloxone protocol was discussed with him as a means of meeting the patient’s medical goals and needs due to the projected efficiency and end goal of buprenorphine depot at the time of discharge, which he consented to.

During the 24 h prior to the induction, the patient had received a total of 48 mg of hydromorphone PO, (3 doses of 16 mg hydromorphone PO) and 10 mg diazepam PO. However, the patient’s CIWA scores were less than 10 prior to this administration and no COWS were completed during this time frame. The patient’s COWS prior to the protocol was 0 and no opiates had been administered for a period of 2 h prior to this induction strategy. 0.1 mg of naloxone intravenously (IV) was administered roughly every 2 min (Table 1). The dose was increased to 0.2 mg IV due to minimal change in COWS score after the administration of the first 2 doses. 0.2 mg IV naloxone was then given three additional times roughly every 2 min, by the fifth dose of IV naloxone, the patient’s COWS was documented at 13, with the patient just approaching moderate withdrawal. This threshold was chosen to ensure that the patient was in moderate withdrawal but was not too uncomfortable to proceed. He had prominent piloerection, lacrimation and rhinorrhea, and was yawning by this point. He was then given a dose of 8 mg buprenorphine-naloxone sublingually, which he tolerated well. His COWS score immediately after receiving the SL dose dropped to 12 and a further reduction of his COWS scores post dose at the 30- and 60-minute mark was documented at 5 and 4. Due to delays in pharmacy processing and dispensing of the medication, the patient received a 300 mg buprenorphine extended-release depot injection 125 min from the time of initiation of the protocol. His COWS score was 0 at this time and expressed satisfaction with the overall patient experience. He was set up with outpatient follow up in two weeks’ time but also self-reported that he was aware of OAT clinics he could connect with for future buprenorphine depot injections, which he intended to stay on. Unfortunately, the patient did not show up to this outpatient appointment and despite repeated attempts to contact the patient via phone for follow up, the patient did not respond.

**Table 1:** Dosage regimen for patient-elected low-dose intravenous Naloxone for rapid buprenorphine induction. Times rounded up to the closest minute

Elapsed Time in Minutes	Medication	COWS (Post Medication when given)
0	0.1 mg naloxone IV	0
2	0.1 mg naloxone IV	1
4	0.2 mg naloxone IV	2
6	0.2 mg naloxone IV	9
8	0.2 mg naloxone IV	13
10	8 mg buprenorphine-naloxone SL	12
30	No medication administration	5
60	No medication administration	4
125	300 mg buprenorphine extended-release	0

## Discussion

Here we have described a case in which a patient with severe OUD and regular fentanyl use was successfully induced on buprenorphine-naloxone and then extended-release injectable buprenorphine through a novel induction protocol of using patient-elected low-dose intravenous naloxone for rapid buprenorphine induction in the inpatient setting. We decided to utilize the protocol to accommodate the patient who wanted to reduce his length of stay in hospital and restart his OAT. Relative to the remaining options of traditional, low-dose, high-dose, or rapid 1-day inductions, this adds to the toolkit as an alternative option for ultra-fast titrations to buprenorphine for hospitalized patients who may otherwise be lost to follow-up. In this case, following induction, the patient was transitioned onto 300 mg of buprenorphine extended-release and expressed satisfaction with the overall process.

This protocol utilized the repeated administration of naloxone at doses starting with 0.1 mg, and escalating to 0.2 mg, to bring about moderate withdrawal for induction. Our use of 0.1 mg is consistent with the initial effective dose of naloxone provided to opioid-dependent patients [20]. It was uptitrated to 0.2 mg when the COWS score remained at one at two minutes post-administration. A total of 0.8 mg of naloxone was successful in eliciting a COWS score of 12 and meeting the requirement to introduce 8 mg of buprenorphine. The hypothesis was that small and incrementally increasing doses of naloxone would accelerate the time to withdrawal in a monitored setting, allowing for a timely administration of buprenorphine-naloxone and accurate assessment of therapeutic effect. This case report, albeit limited to a sample size of one, generates evidence that naloxone can be used in a controlled manner to bring about just enough withdrawal for a standard dose of buprenorphine; all the while avoiding the severe withdrawal associated with large doses of naloxone used to reverse overdose. While withdrawal is incurred, this strategy ensures a rapid buprenorphine initiation as soon as the appropriate COWS threshold is reached, followed by reduction of withdrawal symptoms in a quick and stepwise manner. No signs of opioid toxicity were observed in the patient and the mildly experienced withdrawal (piloerection, lacrimation and rhinorrhea) was thought to be outweighed by the benefits of meeting the patient’s medical goals and initiating his OAT. This technique warrants further refinements with more cases as well as pharmacokinetics and metabolism data for both buprenorphine and naloxone.

The main advantage of this approach is the shortened induction period. The entire induction process from the initiation to the administration of 8 mg of sublingual buprenorphine-naloxone took over a duration of 10 min. This represents a significant improvement from days required from the traditional and low-dose induction methods and the hours from the high-dose and rapid approaches. As of now, the quickest induction methods for short hospital admissions have been 6 and 24 h [21]. As observed with our case, patients often want and need a faster method of treatment initiation than the standard set forth by current protocols. A shorter induction period for hospitalized patients has the potential to greatly improve patient care by removing the barrier of time and the extended period of withdrawal. This can be similarly beneficial for EDs that often face time constraints when initiating interventions for OUD [22].

The inherent limitations of this case report are its sample size of one and that it is unknown whether this protocol is more effective than existing induction protocols. Additionally, it is yet unclear whether this may reliably work for patients who chronically use fentanyl. Accordingly, there is a critical need for further exploration in a larger cohort of patients in ED and inpatient settings to determine the generalizability and relative effectiveness of this protocol. Low-dose intravenous naloxone may offer a feasible way to successfully initiate buprenorphine in those who do not wish to undergo the available induction options or the required period of abstinence from opioids. Moreover, it also carries the potential for use in outpatient and non-medical settings following an overdose as a method to provide patients with more options regarding their treatment. Despite the benefits of buprenorphine, traditional inductions are known to be difficult for patients to tolerate and deter future initiation attempts [23]. In one cohort study of people who use drugs in Vancouver, Canada, less than one-fifth (17.6%) indicated willingness to take buprenorphine [24], with a major reason being the desire to avoid withdrawal. Similarly, a shared limitation of existing methods is the significant length of time required within hospital settings. If the efficacy of this protocol to quickly and effectively pass through withdrawal is confirmed, the findings of this study could potentially decrease the number of patients lost to follow-up and improve the uptake of buprenorphine. Moreover, it may be able to reduce the significant proportion of treatment failure with buprenorphine-naloxone that occurs during the induction phase [25].

## Conclusions

As opioid overdose rates continue to rise in Canada, there is a critical need to reduce barriers to buprenorphine initiation. This case report describes the use of a novel patient-elected low-dose intravenous naloxone for rapid buprenorphine induction protocol to successfully induct a patient with severe OUD onto buprenorphine-naloxone, using small and incremental doses of IV naloxone to accelerate the time to withdrawal. This procedure may be of significant value to mitigate the disadvantages of precipitated withdrawal and significant time consumption in traditional or low-dose inductions. Further research is required to confirm these results and develop an effective protocol for a range of treatment settings.

## Data Availability

No datasets were generated or analysed during the current study.

## Abbreviations

CIWA: Clinical Institute Withdrawal Assessment
COWS: Clinical Opioid Withdrawal Scale
IV: Intravenous
OAT: Opioid agonist treatment
OUD: Opioid use disorder
UDS: Urine drug screen
ED: Emergency department
